# COVID-19 and the denial of structural racism

**DOI:** 10.1177/01410768211011103

**Published:** 2021-08-03

**Authors:** Paramjit Gill, Virinder Kalra

**Affiliations:** 1 Warwick Medical School; 2 department of Sociology

The part played by racism in COVID-19 deaths is receiving considerable attention.^1^ The UK Government Race Disparity Unit report released on 22 October 2020^2^ with Dr Raghib Ali, senior advisor, stating that they ‘… ruled out structural racism as a driving factor behind BAME people’s poorer health outcomes after catching Covid, saying there was “no evidence” to support the theory’. Five days later, the UK Labour Party released a new report on the disproportionate impact of the coronavirus crisis on minority ethnic communities^3^ stating structural racism had played a part in the disproportionate impact of COVID-19 on Black, Asian and Minority Ethnic communities. It is clear that racism has become an ideological battleground in which the present UK government seeks to deny its existence, while using ‘race' as a classificatory category. Further evidence for this contradictory stance is the equalities minister questioning the validity of unconscious bias training and the application of critical race theory (which highlights structural racism and inequality). Another concern is over the selection of David Goodhart as a commissioner of the Equality and Human Rights Commission. Goodhart is a supporter of the Home Office's hostile environment policy and someone who has advocated for ‘white self-interest'.^4^ This appointment is yet another example of a government relying on race to maintain and promote systems of oppression while simultaneously denying the full ramifications of structural racism.^5^

In the meantime, the statistics on inequalities in COVID-19 mortality remain troublesome. We acknowledge that this is a complex area but even here, the new mantra of diabetes, overweight and hypertension is foregrounded to highlight how it is individual behaviour (always invoked alongside moves of group reductionism through the narrative of *culture*) that is at fault. Where racism would indicate that the disproportionate deaths within ethnic minorities are a summation of concentration in over crowded housing, high levels of employment in front-line services, occupation in high-risk categories and systematic abuse (at the hands of state institutions and individuals),^6^ the focus of this government is only on ‘underlying’ medical conditions – racism is not mentioned at all in its report!^2^ Yet the racial and ethnic health inequalities that COVID-19 has exposed are longstanding and deeply rooted in countries such as the UK and US.^7,8^

There is a long legacy of commissioned reports on inequalities beginning with The Black Report that highlighted the marked inequalities between the social groups.^9^ Subsequent reports confirmed and highlighted that differences between the groups were widening and entrenched.^10–12^ The causes of this disparity tend to focus either on individual/community factors – behavioural or biological influences, for example – or on structural explanations: particularly the health impact of the socioeconomic deprivation experienced disproportionately by people from minority ethnic groups. The influence of racism on these patterns has often been underestimated or ignored.

What are the underlying mechanisms for this? Research shows that racism-related adversity and stressors result in increased activity in the sympathetic nervous system (with rise in blood pressure) and the hypothalamic–pituitary–adrenal axis (e.g. elevated cortisol levels); when these physiological stress responses are repeatedly activated, they lead to increased risks for an array of health conditions.^13–15^

The role of structural racism in worsening health outcomes for ethnic minorities should not be in dispute. The debate should not be manipulated to avoid government facing hard truths. The absence of strategies to address racism and health inequalities has been exposed by a new virus^1,16^ and, while that remains the case, it is the duty of health professionals to speak up for patients by demanding an acceptance of the central role of racism in determining the health of ethnic minorities.

## Declarations

**Ethics approval:** Not applicable

**Guarantor:** Paramjit Gill.

**Contributorship:** PG conceived the idea that was developed with VK. Both authors approved the final version.
